# State Trends of Cannabis Liberalization as a Causal Driver of Increasing Testicular Cancer Rates across the USA

**DOI:** 10.3390/ijerph191912759

**Published:** 2022-10-05

**Authors:** Albert Stuart Reece, Gary Kenneth Hulse

**Affiliations:** 1 Division of Psychiatry, University of Western Australia, Crawley, WA 6009, Australia; 2 School of Medical and Health Sciences, Edith Cowan University, Joondalup, WA 6027, Australia

**Keywords:** testicular cancer, drugs, cannabis exposure, gene-environment interaction, pathways and mechanisms

## Abstract

Background. The cause of the worldwide doubling-tripling of testicular cancer rates (TCRs) in recent decades is unknown. Previous cohort studies associated cannabis use with TCR including dose–response relationships but the contribution of cannabis to TCRs at the population level is unknown. This relationship was tested by analyzing annual trends across US states and formally assessed causality. Four US datasets were linked at state level: age-adjusted TCRs from Centers for Disease Control Surveillance Epidemiology and End Results database; drug use data from annual National Survey of Drug Use and Health including 74.1% response rate; ethnicity and median household income data from the US Census Bureau; and cannabinoid concentration data from Drug Enforcement Agency reports. Data was processed in R in spatiotemporal and causal inference protocols. Results. Cannabis-use quintile scatterplot-time and boxplots closely paralleled those for TCRs. The highest cannabis-use quintile had a higher TCR than others (3.44 ± 0.05 vs. 2.91 ± 0.2, mean ± S.E.M., t = 10.68, *p* = 1.29 × 10^−22^). A dose–response relationship was seen between TCR and Δ9-tetrahydrocannabinol (THC), cannabinol, cannabigerol, and cannabichromene (6.75 × 10^−9^ < *p* < 1.83 × 10^−142^). In a multivariate inverse probability-weighted interactive regression including race and ethnic cannabis exposure (ECE), ECE was significantly related to TCR (β-estimate = 0.89 (95%C.I. 0.36, 2.67), *p* < 2.2 × 10^−16^). In an additive geospatiotemporal model controlling for other drugs, cannabis alone was significant (β-estimate = 0.19 (0.10, 0.28), *p* = 3.4 × 10^−5^). In a full geospatial model including drugs, income and ethnicity cannabinoid exposure was significant (cannabigerol: β-estimate = 1.39 (0.024, 2.53), *p* = 0.0017); a pattern repeated at two spatial and two temporal lags (cannabigerol: β-estimate = 0.71 (0.05, 1.37), *p* = 0.0.0350; THC: β-estimate = 23.60 (11.92, 35.29), *p* = 7.5 × 10^–5^). 40/41 e-Values > 1.25 ranged up to 1.4 × 10^63^ and 10 > 1000 fitting causal relationship criteria. Cannabis liberalization was associated with higher TCRs (ChiSqu. = 312.2, *p* = 2.64 × 10^−11^). Rates of TC in cannabis-legal states were elevated (3.36 ± 0.09 vs. 3.01 ± 0.03, t = 4.69, *p* = 4.86 × 10^−5^). Conclusions. Cannabis use is closely and causally associated with TCRs across both time and space and higher in States with liberal cannabis legislation. Strong dose–response effects were demonstrated for THC, cannabigerol, cannabinol, cannabichromene and cannabidiol. Cannabinoid genotoxicity replicates all major steps to testicular carcinogenesis including whole-genome doubling, chromosomal arm excision, generalized DNA demethylation and chromosomal translocations thereby accelerating the pathway to testicular carcinogenesis by several decades.

## 1. Background

### 1.1. Incidence

Testicular cancer (TC) is the most common cancer in males aged 15–44 years, and responsible for more years of life lost than any other adult cancer [1]. In recent decades, testicular cancer rates (TCRs) have unexplainably risen two- to three- fold in many nations [2,3,4].

Testicular germ cell tumours (TGCT) comes in many variants. In males aged 15–44 years it is usually of either of the seminoma (50%) or non-seminoma (40%) variety, with 10% being of mixed subtype [1,4,5,6,7]. Non-seminoma germ cell tumours (NSGCT) can be of either the embryonal or teratoma or yolk sac or choriocarcinoma varieties depending on whether embryonal tissues or extraembryonal tissues are developed [8]. Germ cell neoplasia in situ (GCNIS) is believed to be the tissue of origin of seminoma; GCNIS or seminoma is believed to be the tissue of origin of embryonal carcinoma which is believed to be the source of extraembryonic (yolk sac or choriocarcinoma) and somatic (teratoma) lineages [8].

Twenty-fold variation in TCR have been documented around the world [1,2,3,4,8] with two-fold variations across the same continent [3], and even within the same country as geographic clusters [9].

SEER*Explorer is an online data portal maintained by the Centers for Disease Control and the National Cancer Institute. It allows online checking of many features of cancer epidemiology such as short and long term trends, age-, sex- and ethnic- specific rates in both tabular and graphical formats. Data from SEER*Explorer reveals that the age-adjusted rate of testicular cancer in US males for all ages and all stages rose 83.45% from 3.4415 to 6.3136/100,000 1976–2017 [10]. When considering the change in the peak age incidence of males 15–39 years the rate rose 92.14% from 6.2922 to 12.091/100,000 1975 to 2017 which represented an annual percent change of 3.31% 1975–1986 and 0.7424 1987–2017. The cause of these concerning rises in TCR is unknown.

### 1.2. Risk Factors

Many risk factors have been described for TGCT including cryptorchidism, testicular dysgenesis syndrome including congenital urogenital anomalies including hypospadias, infertility, inguinal hernia repair, having a previous TGCT, having a close family relative with TGCT (eight to ten-fold for a brother and four to six- fold for a father), exposure to three organochlorines (dichlorodiphenyldichloroethylene, cis-nonachlor and trans-nonachlor) and certain occupational groups such as firemen and aircraft workers [1,4,6]. Endocrine disruption such as maternal bleeding, low birthweight, twinship, short gestation, tall stature, first position in the sibship and small sibship, Downs syndrome and Klinefelters syndrome are also implicated [4,5], as is the use of cannabis both through gestational exposure and adult use [4,11].

All four studies to have examined the association between TC and cannabis use have found a positive relationship [12,13,14,15]. Dose response relationships have been demonstrated for frequency of use [13,15], for long term use [15], for total dose exposure (more than 50 times) [12] and the age of first onset (less than or older than eighteen years of age) [13]. Where the relationship to different tumour histiotypes was examined the risk was confined to non-seminomatous germ cell tumours and was not seen for seminoma. In meta-analysis cannabis use was shown to provide an elevation of risk for non-seminoma of 2.59 (95%C.I. 1.60–4.19) [2]. These findings suggest that cannabis exposure through personal use likely increases incidence of non-seminomatous germ cell tumours but not seminoma: notwithstanding, in utero exposure may remain a risk factor for both.

A significant number of women are using cannabis whilst pregnant across the USA and this number is rising [16,17,18]. Nationwide 161,000 American women were estimated to have used cannabis whilst pregnant in 2017 [19]. A 2018 study found that 24% of Californian pregnant teenagers smoked cannabis whilst pregnant [20], while 69% of Colorado cannabis dispensaries recommended cannabis to pregnant clients, sometimes for symptoms associated with pregnancy [21]. Such data may be relevant to what is generally believed to be the origins of TGCT during antenatal development [1,3,5,6,7]. This increased use is likely driven both by liberal legislation which allows access to cannabis both for personal and/or medical use and the widespread popular misperception of cannabis benignity and “soft drug” status.

The testis (and ovary) are unique amongst body tissues since the gonads are believed to be the only site of long lived pluripotential germ cells [4]. Since their relatively unmethylated epigenomic state make them particularly susceptible to genotoxic and epigenotoxic insults [4,22] they may represent a site of unique vulnerability to the effects of environmental intoxicants and mutagens, which may explain the relative susceptibility to testicular carcinogenesis as opposed to carcinogenic effects in other tissues. TGCT’s are known to be highly heritable [23]. One moderate penetrance allele at checkpoint kinase 2 (CHEK2) and 78 low penetrance alleles together confer 44% of the familiar risk [23]. Three somatic mutations implicated in TGCT include KIT, NRAS and KRAS. TP53 mutations confer platinum resistance [23].

### 1.3. Hypotheses

For several reasons therefore it becomes reasonable to examine in some detail the epidemiological associations of cannabis use and TCRs using as an experimental environment the variance across time and space between the various US states. This approach has several advantages including the ready and public availability of required data including testicular cancer rates, cannabinoid and other substance exposure and ethnographic and income data, and that the use of cannabis across many US States has changed rapidly in recent years. The main questions addressed in this epidemiological study are: (1) “Does the previously described relationship between cannabis and TC survive multivariable adjustment?”; (2) “Is this effect strong enough to drive the remarkable rise in TCR?”; (3) “What are the effects of cannabis legalization on the TCR?”; and (4) “Does the cannabis-TC relationship satisfy the quantitative criteria of causal inference? [24,25]”.

Whilst the qualitative criteria of causal inference are well known and were eloquently stated in 1965 by A.B. Hill [26] more recent studies have defined important quantitative criteria which also apply to potentially causal relationships and relate to both known and unknown confounding covariates. Measured covariates are optimally controlled by inverse probability weighting of multivariable models. The maximal effect of unmeasured (also called “uncontrolled’) confounders can be quantified by the use of E-values which effectively sets limits on what the collective contribution of confounders not considered by the study analysis can be. These important technical refinements are described further below.

## 2. Methods

### 2.1. Data sources and Record Linkage Procedure

Data linkage occurred at the state level for all datasets. USA state-based data on age-adjusted TCRs for patients aged 15 to 60 years was taken from the Centres for Disease Control (CDC) National Program of Cancer Registries (NPCR) and Surveillance Epidemiology and End Results (SEER) Incidence File from the US Cancer Statistics Public Use Database Submission 2001–2017 downloaded via the SEERStat software [27]. National rates including ethnic and age categorized data were taken from the SEER*Explorer website [10]. Drug use data for the period 2003–2017 was obtained from the Restricted Use Data Analysis System (RDAS) of the Substance Abuse and Mental Health Data Archive (SAMHDA) of the National Survey of Drug Use and Health (NSDUH) from the Substance Abuse and Mental Health Services Administration (SAMHSA) [28]. The drugs of interest were last month cigarette use (Cigarettes), last year Alcohol Use Disorder (AUD), last month cannabis use (Cannabis), last year analgesic misuse (Analgesics) and last year cocaine use (Cocaine). Drug use rates were for both sexes combined. The combined sex exposure rate was the mean of the (male + female) rates. In all cases the use rate amongst males was higher than the use rate amongst females. Median household income and ethnicity data was downloaded from the US Census bureau via the tidycensus package in R [29]. The ethnicities of interest were Caucasian-Americans, African Americans, Hispanic-Americans, American Indians/Alaskan Native (AIAN) -Americans and Native Hawaiians/Pacific Islander (NHPI) -Americans. The concentration of cannabinoids was taken from publications of the Drug Enforcement Agency [30,31,32]. Data relating to the legal status of cannabis was derived from an internet search [33]. Missing data were filled by temporal kriging (temporal mean substitution). Data from the four datasets was combined by state and by year.

### 2.2. Derived Data

A variable called “mrjmdays” on the SAMHDA RDAS data file lists the number of days of cannabis used last month as a categorical variable with categories at 0, 1–2, 3–5, 6–19 and 20–30 days last month use. It can be cross-tabulated by ethnicity at the national level to derive an ethnic score for intensity of cannabis use for each year of the NSDUH survey. This can then be multiplied by the state rate of last month cannabis use to derive a state-based cannabis use index for that ethnicity. This score was then multiplied by the tetrahydrocannabinol (THC) concentration in that year to derive an index of ethnic THC exposure by state. The intensity of cannabinoid exposure is clearly of great relevance to considerations of genotoxicity as not only the fraction of the population with any exposure, but the depth of the exposure of that population is likely to be highly pertinent to the degree of genotoxic outcomes which may be expected to occur (see also the Discussion section). The state-based exposure to cannabinoids was derived by multiplying the last month cannabis use for that state by the cannabinoid concentration in federal seizures. Cannabis use quintiles were calculated by dividing the states into equal quintiles of cannabis use for each year of the NSDUH and then combining these annual quintiles across years.

### 2.3. Statistics

Data was processed using R version 4.0.2 and R-Studio 1.3.1093 in October 2020. Data were manipulated using dplyr from the tidyverse suite of packages [34]. Graphs were drawn in ggplot2 [35] and lattice [36] and maps were drawn in ggplot2 and sf [37] using RColorBrewer [38]. Point data are listed as mean ± standard error of the mean. Data were log transformed guided by the Shapiro–Wilks test. Initial regression models were reduced by manual serial deletion of the least significant term according to the classical method of model reduction. Linear regression was performed in R-Base. Inverse probability weights were derived for cannabis exposure as a function of all other substance use [39]. Mixed effects regression was performed with the package nlme [40] with State as a random effect. Robust regression was performed with the survey package again using state as the identifying variable [41]. Mixed effects and robust regression models were performed with inverse probability weights in all cases.

#### 2.3.1. Spatial Regression

Spatiotemporal regression was performed in R-package splm [24,42] using using geographic (State) weights lists compiled in spdep [25] and edited as shown. The spatial dependencies were determined by the edge and corner spatial relationships in so-called “queen relationships” by analogy with the moves of the Chess piece of the same name. “spdep” (Spatial Dependencies) is a specialized R-package dedicated to the formulation and computation of spatial relationships between regions. The centroids of each region is taken by default from the larges polygon for each region. Links represent geospatial relationships rather than any other metric.

Spatial regression was performed using the spreml (spatial panel random effects maximum likelihood) function in splm [43] initially with the full error structure (sem2srre) of spatial errors according to Kelejian, Kapoor and Prucha (KKP) [44], serial autocorrelation in the error structure with random effects and spatial lagging. KKP errors are appropriate where reasons exist for considering that both the exposures and the outcomes are likely to be spatially autocorrelated. Given the spatially and temporally orchestrated nature of the US cannabis legalization campaign and existence of cannabis as an established risk factor for testicular cancer it seemed highly likely that not only the exposure but also the outcome was likely to be spatially autocorrelated. Final model specification was chosen from the significant parameters from the full model as suggested by the package authors [45].

#### 2.3.2. Multiple Regression Techniques

A variety of regression types were used for the following reasons. Straightforward linear regression was used for overall analysis where a straightforward overall effect was of interest. Mixed effects models include both fixed and random effects and take account of the state-by-state repeated measures autocorrelative structure in the data and account for recurrent taking of samples from the same spatial units. Robust regression techniques allow for the use of robustified regression applications to the data structure. Geospatial analysis allows for the consideration of the data in their native real-world spatiotemporal situation which in this context is highly relevant as the liberalization of cannabis legalization is known to have occurred in a systematic fashion from the west coast eastwards and is thus intrinsically spatially autocorrelated. Spatiotemporal analysis formally accounts for such spatial and temporal autocorrelative structures. Both mixed effects and robust regression can be inverse probability weighted which allows their results to be considered in a formal causal framework. Both mixed effects and spatial models include model standard deviations in their final model structures which allows the calculation of E-values from these model types. Hence, the use of more sophisticated forms of regression techniques integrates the present regression analyses with the major techniques of causal inference and cross-validates the major results between the various regression platforms.

### 2.4. E-Values

e-Values were calculated from package EValue [46]. The E-Value (or Expected Value) quantifies the degree of association required of some unmeasured hypothetical confounding variable with both the exposure of concern and the outcome of interest to explain away an apparently causal effect. It is computed on the relative risk scale. It thus sets quantitative limits on the strength of association required of unmeasured extraneous variables external to the measured covariates included in the study and thereby places strong parametric limits on the plausibility of extraneous unmeasured covariates as explanations for the observed effects. The value of 1.25 is typically taken as the minimum level for a putatively causal relationship [47].

All *t*-tests were two tailed. *p* < 0.05 was considered significant.

### 2.5. Data Availability Statement

All data including software code has been made freely available on the Mendeley data repository and may be found at this URL http://doi.org/10.17632/ttzb9xvb4v.1 (accessed on 18 October 2020).

### 2.6. Ethics

This study has received ethical approval from the University of Western Australia Human Research Ethics Committee and was accepted on 7 January 2020 RA/4/20/4724.

## 3. Results

### 3.1. Data

Data from the SEER*Explorer website indicates that 80.09% of TC cases occur in the age range 15–60 years. This is also the age range for which ethnicity data is most complete. For these reasons the age range 15–60 years formed the study group of interest. As also shown on the SEER*Explorer website the age peak for testicular cancer is 30–34 years of age.

State age-adjusted TCRs were downloaded from the SEER databases as indicated via the SEERStat software. In the period 2001–2017 there were 850 potential data points for the fifty states which were filled by 837 TCR’s. 13 missing values from a vector of 850 datapoints equates to a rate of 1.53% missing data. Missing data were filled by temporal kriging. Data are shown in Supplementary Table S1 with kriged data highlighted.

**Figure 1:**
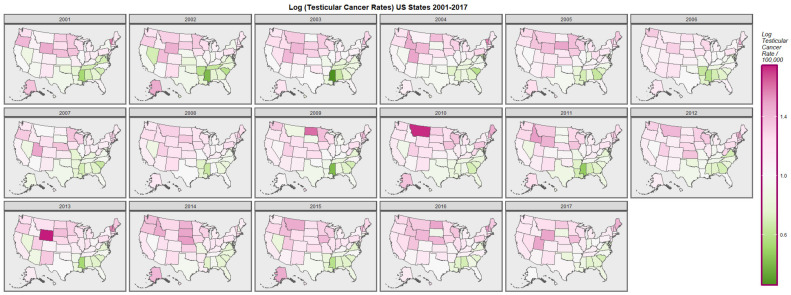
Map-graph of log(testicular cancer incidence rates) across USA by state and year.

**Figure 2:**
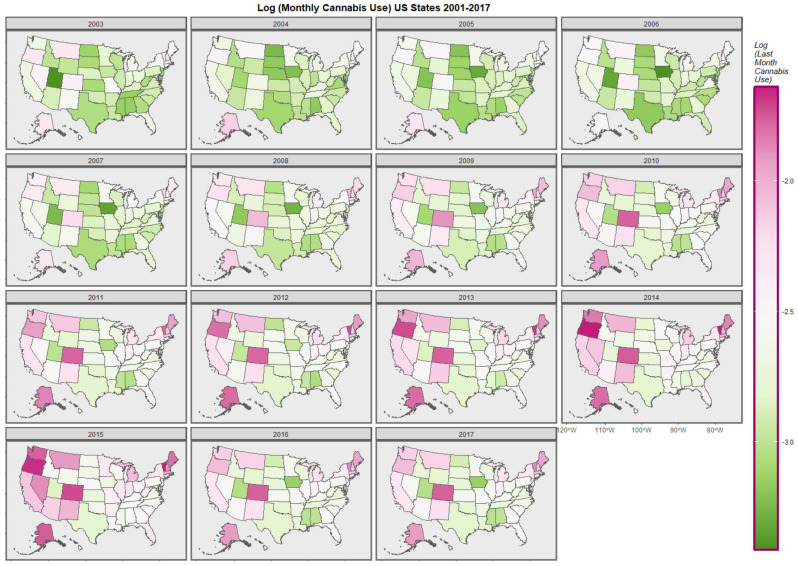
Map-graph of log(last month cannabis use rates) across USA by state and year.

Figure 1 graphically maps the log (TC) rates for USA States across years. Figure 2 shows a comparable map-graph of the log of last month cannabis use over time across almost the same period, 2003–2017. Since this is the period for which all the drug use data was available this became the period of analysis.

### 3.2. Bivariate Analysis

**Figure 3:**
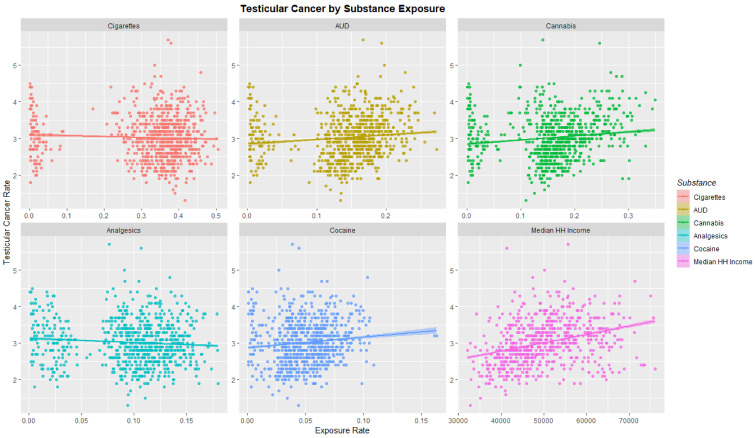
Testicular cancer incidence rates by substance exposure. Substance exposure is listed as the fraction of the population reporting the applicable exposures. Median household income is reported as annual income in US dollars.

The TCR was charted against substance exposure as shown in Figure 3. Substance exposure is illustrated as a fraction of the population reporting the exposure. Median household income is shown as median annual salary in US dollars. Strong positive upward trends are shown with AUD, cannabis and cocaine exposure and with median income.

**Figure 4:**
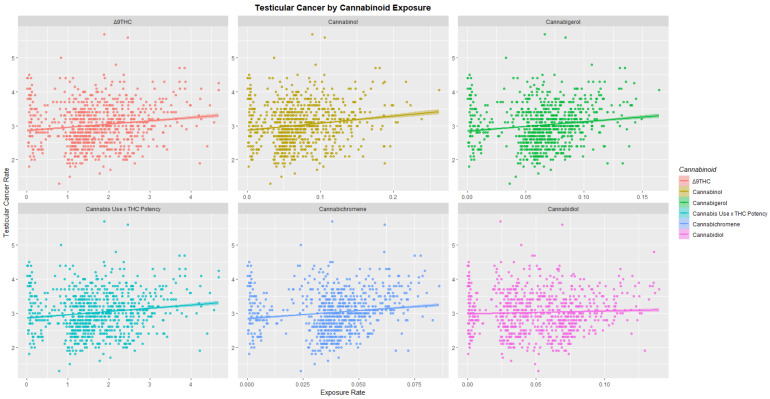
Testicular cancer incidence rates by cannabinoid exposure.

When the USA TCR was charted against exposure to various national trends in cannabinoids THC, cannabinol, cannabigerol, cannabichromene and cannabidiol as shown in Figure 4 positive associations were shown.

**Figure 5:**
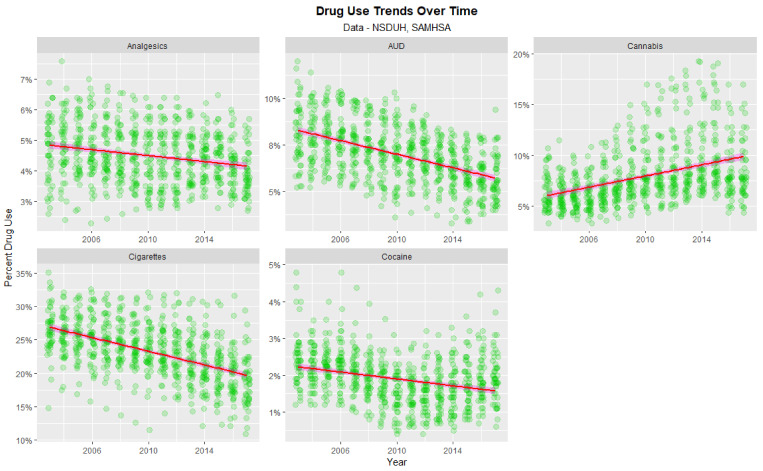
Time course of drug use across USA.

Important to the consideration at hand is the time trend of drug exposure. As shown in Figure 5 the rate of analgesic abuse, AUD, cigarette use and cocaine use fell across this period; only the use of cannabis rose across this period.

### 3.3. Effect of Cannabis Legal Status on Drug Use

**Figure 6:**
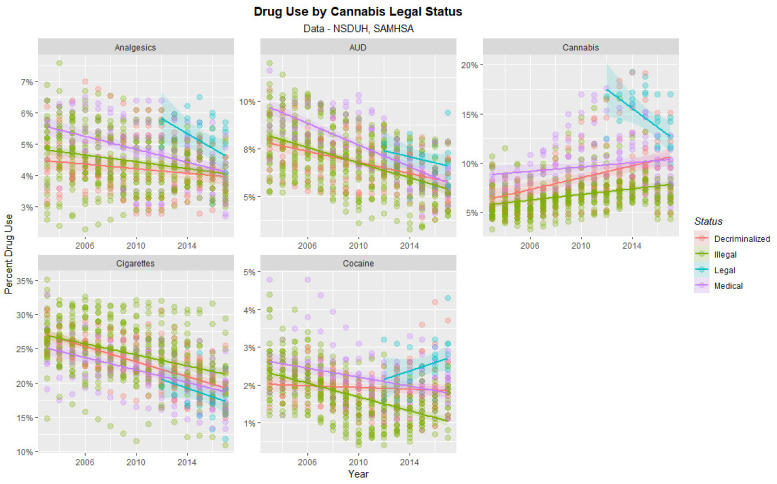
Time course of drug use across USA by cannabis legal status—scatterplots.

The time dependent trajectory of drug use by cannabis legal status is shown in Figure 6. Significant trends are shown for states with legal cannabis particularly in relation to increases in cocaine and initial elevations and subsequent reductions in analgesics and cannabis.

**Figure 7:**
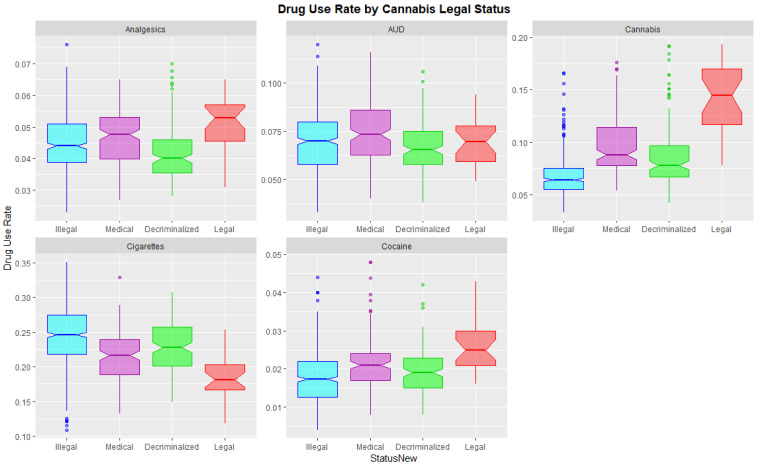
Substance use across USA by cannabis legal status—boxplots with aggregated time. Note that where the notches on the boxplots do not overlap this signifies a statistically significant difference.

Figure 7 shows this data as boxplots aggregated across years and States. Where the notches of the boxes do not overlap this indicates statistically significant differences. Legalization is associated with significantly higher cannabis, cocaine and analgesic use and lower cigarette use.

Supplementary Table S2 shows the State cannabis use rates which were divided into quintiles in each year.

### 3.4. Cannabis Use Quintiles

**Figure 8:**
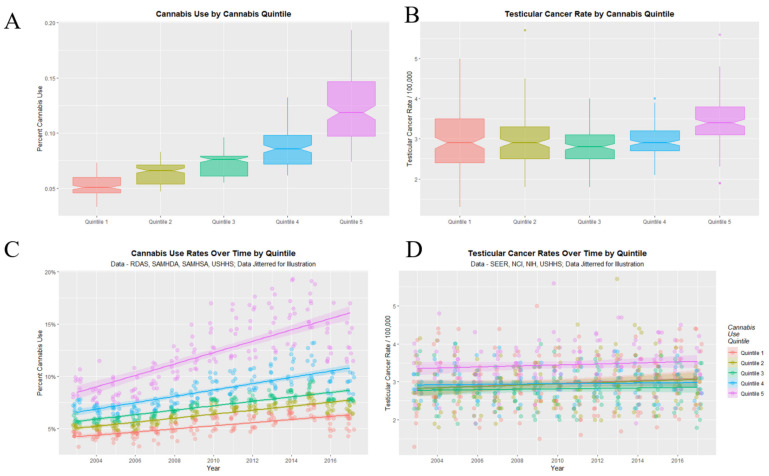
Cannabis use and testicular cancer incidence rates by quintiles of cannabis use. (**A**,**C**) cannabis use. (**B**,**D**) testicular cancer rates. (**A**,**B**) boxplots. (**C**,**D**) scatterplots with regression lines. Note that where the notches on the boxplots do not overlap this signifies a statistically significant difference.

Figure 8 graphs the cannabis use (A,C) and TCR’s (B,D) as boxplots (A,B) and scatterplots (C,D). Categorization of cannabis use by quintiles neatly stratifies cannabis use both as scatterplots and boxplots (panels A,C). Importantly the highest quintile of cannabis use is also the highest quintile of TCR (panel D). The fifth cannabis use quintile line is clearly elevated in TCR relative to the lower quintiles across all years (panel D). Considering the boxplot shown in panel B one notes that the notches of the lower four quintiles are all overlapping so they are not significantly different. However, the notch of the fifth quintile is very much higher than any of the others. This clearly indicates an abrupt step effect from the fourth to the fifth quintile.

### 3.5. Dichotomized Quintile Data

**Figure 9:**
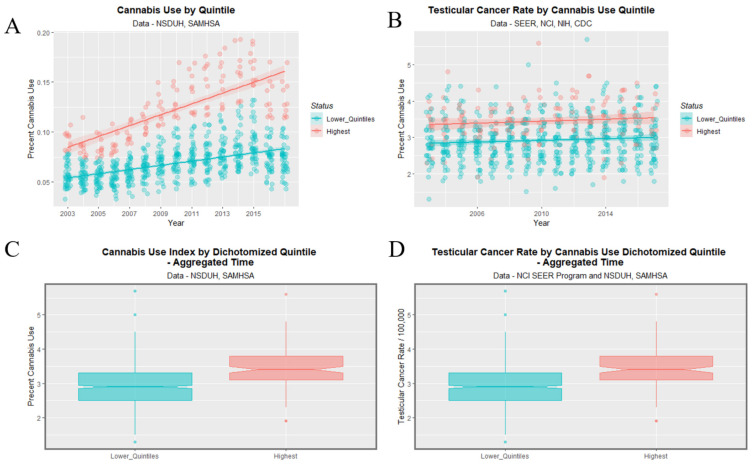
Cannabis use and testicular cancer incidence rates by dichotomized quintiles of cannabis use. Dichotomy contrasts highest cannabis use quintile with the lower four quintiles. (**A**,**C**) cannabis use. (**B**,**D**) testicular cancer rates. (**A**,**B**) scatterplots with regression lines. (**C**,**D**) boxplots.

Figure 9 shows this data dichotomized between the highest quintile and the four lower quintiles. One readily observes that the highest quintile is higher than the others across the time course for both cannabis use and TCR. The lack of overlap with the notches on the boxplots on the two lower panels demonstrates the highest quintile had significantly higher aggregated cannabis use and TCRs.

The mean TCR in the lower quintiles is 2.915 ± 0.024/100,000 and that in the higher quintiles is 3.442 ± 0.046/100,000 (mean ± S.E.M., t = 10.679, df = 260.22, *p* = 1.29 × 10^−22^).

**Figure 10:**
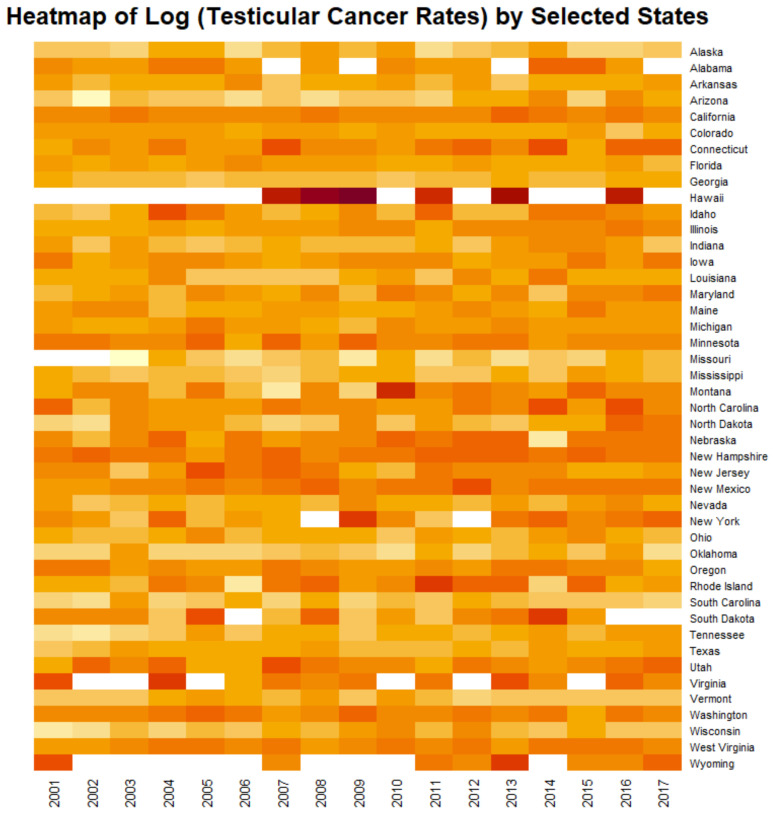
Heatmap of testicular cancer rates by state. Note Hawaii near the top which is an obvious standout hotspot.

Figure 10 shows heatmap of the age adjusted log (TCR’s) by state. The very hot spot in Hawaii for all years stands out prominently.

### 3.6. Multiple Regression

#### 3.6.1. Linear Regression

Table 1 shows linear regression results for the TCR against time, cannabis use, the time: cannabis use interaction, and additive model with other drugs and by quintiles. One notes that cannabis use is highly significantly related across the whole population to the TCR both when regressed alone (β-estimate = 0.47 (95%C.I. 0.34, 0.59), *p* = 7.50 × 10^−13^) and when considered along with time (β-estimate = 0.47 (0.34, 0.59), *p* = 7.50 × 10^−13^). Importantly in an additive model with the other four drugs cannabis use is highly significant (β-estimate = 0.45 (0.32, 0.57), *p* = 7.24 × 10^−12^).

Table 1 also gives the slopes of the regression lines shown in Figure 3 and Figure 4. High *p*-values are noted especially for THC, cannabigerol, cannabichromene and cannabinol.

One notes here that the time: highest quintile interaction is highly significant for the dichotomized quintile analysis (β-estimate = 0.17 (0.14, 0.21), *p* = 7.24 × 10^−21^).

#### 3.6.2. Mixed Effects Regression

The results of inverse probability weighted mixed effects regression appear in Table 2. Here, cannabis alone is highly significantly related to TCR (β-estimate = 0.16 (0.15, 0.18), *p* = 1.70 × 10^−75^) and in an additive model with other drugs cannabis is also highly positively related (β-estimate = 0.15 (0.14, 0.17), *p* = 1.14 × 10^−68^). In the final 4-Way interactive model with income cannabis appears in six terms and is strongly and independently significant (β-estimate = 4.25 (2.95, 5.54), *p* = 1.70 × 10^−10^).

#### 3.6.3. Robust Regression

Final regression models from inverse probability weighted robust regression are presented in Table 3. Cannabis and ethnic cannabis exposure are again noted to be highly statistically significant. Ethnic effects are also noted to be significant. Further detailed dissection of ethnic effects by robust regression is left to a subsequent manuscript.

Supplementary Figure S1 presents the 50 states for which TCR data is available. Panel A presents the 2017 cannabis use data and panel B illustrates the 2017 TCR data.

#### 3.6.4. Geospatial Regression

**Figure 11:**
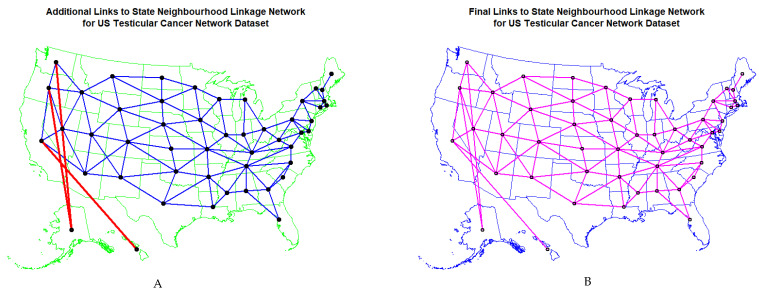
Map of geospatial neighbour links use din spatial regressions (**A**) edited and (**B**) final.

Figure 11 shows (A) the edited and (B) the final geospatial links which were derived from the software. Details relating to the manner in which these spatial links were calculated are provided in the Methods section.

These spatial weights were used in geospatiotemporal regression models. The results of increasingly complex final spatial models are presented in Table 4. Terms including cannabis, cannabigerol, THC and ethnic THC exposure continue to be highly significant as indicated. One notes that in an additive model cannabis exposure alone was highly and independently significant and was the sole remaining term after model reduction (β-estimate = 0.19 (0.10, 0.28), *p* = 3.42 × 10^−5^).

### 3.7. E-Values

These various data are associated with e-Values some of which are presented in Table 5. Table 6 lists E-Value estimates and minimal e-Values in descending order. Note that in order to place both lists in consecutive descending order it has been necessary to break the connection between the e-Value pairs. e-Value estimates range from 1.60 to 8.61 × 10^81^ (median 3.68, IQR 2.48, 1.28 × 10^5^) and all exceed 1.25 which has been proposed in the literature as the cut-off level indicating likely causality [47]. 40/41 minimum e-Values are noted to be higher than 1.25 and range up to 1.40 × 10^63^ and include 26 greater than 2.0 and 10 greater than 1000. The median minimum e-Value is 2.76 (IQR 1.88, 2790).

### 3.8. Legalization

#### 3.8.1. Cannabis Legal Status

**Figure 12:**
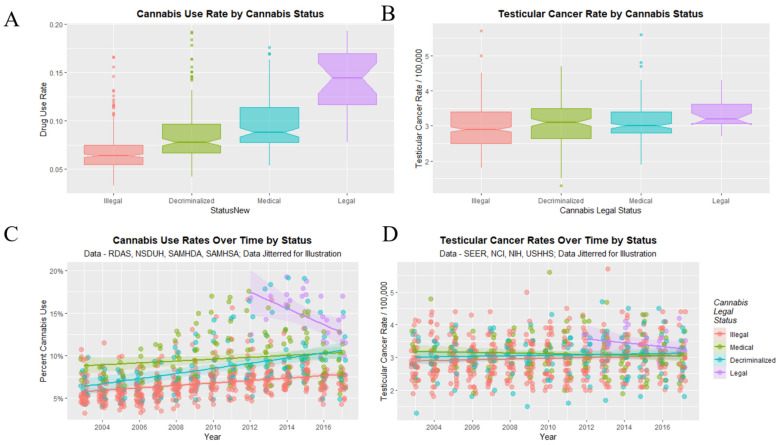
Cannabis use and testicular cancer incidence rates by cannabis legal status. (**A**,**C**) cannabis use. (**B**,**D**) testicular cancer rates. (**A**,**B**) boxplots. (**C**,**D**) scatterplots with regression lines.

Finally, it remained to consider the impact of cannabis legalization on the TCR. As shown in Figure 12A there are elevations in cannabis use in association with the relaxation of cannabis laws. Figure 12C shows elevations from the start of legal cannabis and increases across years with decriminalization. Cannabis use rates in Figure 12A,C appear to be reflected in TCR’s in panels Figure 12B,D.

TCR’s under the illegal, decriminalized, medical and legal paradigms were 2.956 ± 0.029, 3.064 ± 0.053, 3.096 ± 0.047 and 3.361 ± 0.086 (mean ± S.E.M./100,000) respectively. A significant trend was found (Chi Squ. = 312.2, df = 164, *p* = 2.63 × 10^−11^).

#### 3.8.2. Dichotomized Cannabis Legal Status

**Figure 13:**
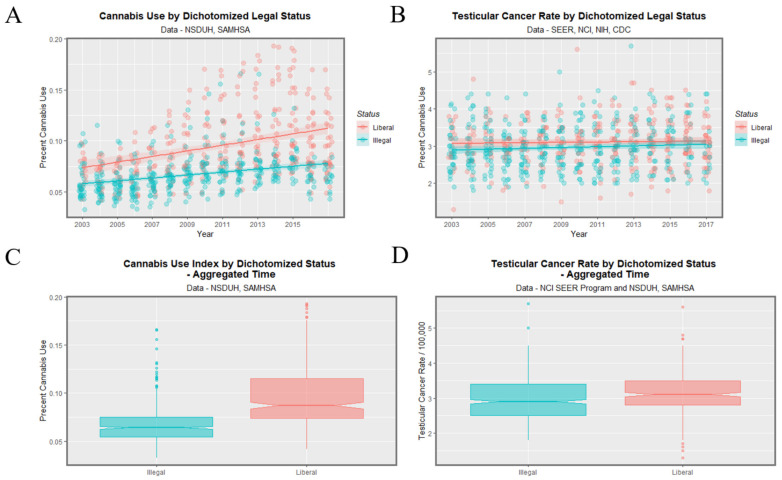
Cannabis use and testicular cancer incidence rates by dichotomized cannabis legal status. Dichotomy contrasts states where cannabis is illegal vs. others. (**A**,**C**) cannabis use. (**B**,**D**) testicular cancer rates. (**A**,**B**) scatterplots with regression lines. (**C**,**D**) boxplots with notches.

Data may be dichotomized by contrasting illegal states with more liberal ones as shown in Figure 13. Higher cannabis use rates in panels A, C seem to be reflected in higher TCR’s in panels B, D. The notches pertaining to TCR in Figure 13D do not overlap. The TCR in illegal states was 2.957 ± 0.029 whereas that in liberal states was 3.104 ± 0.033 (t = 3.3566, df = 696.82, *p* = 8.32 × 10^−4^).

States with legal cannabis had a higher TCR than others (3.3607 ± 0.0861 vs. 3.0073 ± 0.0229, t = 4.6865, df = 32.218, *p* = 4.86 × 10^−5^).

Table 7 lists the applicable *p*-values at linear regression and finds many highly significant values all in the expected direction. The relevant e-Values pertaining to these data are shown in the lower portion of Table 5 where all minimum e-Values are noted to be above the critical threshold value of 1.25 [47].

## 4. Discussion

### 4.1. Main Results

Analysis of study data using a variety of different techniques indicate that cannabis use is closely associated with TCR across both years and States with this association satisfying the quantitative criteria of causal inference. This relationship is strengthened by consideration of ethnic THC exposure, and by consideration of cannabinoid exposure from agents such as THC and cannabigerol. US State TCRs were related to cannabis legal status, with TCRs higher in States with liberal cannabis legislation and lower in those with legal restrictions to use.

### 4.2. Biological and Mechanistic Considerations: Cannabis and TC

A brief review of the mechanistic basis of cannabinoid related testicular carcinogenic pathways is relevant to this epidemiological discussion to aid general understanding and appreciation of the effect and to directly address the ‘biological plausibility’ clause of the Hill criteria which is one of the qualitative means of establishing causal relationships [26]. This section will consider the known pathobiology of testicular oncogenesis, the known genotoxic pathophysiology of cannabinoids and demonstrate the manner in which these two sets of cancerogenic processes closely coincide.

The biology of non-seminomatous germ cell tumour (NSGCT) is being described in considerable detail which is leading to important treatment developments [1,4,8]. This is of great significance not only in delineating more effective treatment but also because it enable the identification of mechanistic pathways by which environmental intoxicants such as cannabis can act as an antecedents for TC.

### 4.3. TGCT Pathobiology

It has been shown that TC generally develops from antenatal genomic perturbations to GCNIS which undergo transformation after the hormonal surge of adolescence [1,4,8].

TGCT are characterized mainly by copy number variants (CNV’s) and chromosomal aberrations. Single nucleotide variants (SNV’s) are quite rare and average only 0.5/MB [4,8]. The pathogenic pathway to TGCT development is known to begin with one or two whole genome doubling events so that the normal karyotype of 2 N rises to 4 N and sometimes 8 N. This is thought to occur through dysfunction of the mitosis/meiosis switch [4]. Spermatocytes normally have haploid ploidy at 1 N. From 4 N malignant cells whole chromosomes and whole arms of chromosomes are progressively lost due to the genomic instability of polyploidy and genomewide demethylation. Seminomas have 30–50 lost chromosomal arms and NSGCT have 50–70 lost chromosomal arms [8]. Seminomas have a median of 3.1 N and NSGCT have a median of 2.8 N [8].

TGCT’s invariably have gains of the short arm of chromosome 12 [4,5,8,48,49]. In 87% of cases this is as an isochromosome and in other cases it is as a gene amplification event. This is important to tumour biology as the short arm of chromosome 12 is a stem cell locus [5]. Isochromosome 12 p ((i)12 p) arises from an aberrant centromeric anaphase division in a tetraploid (reduplicated) genome [4]. Tumours have been described harbouring four chromosome 12′s and an isochromosome 12 p [4]. (i)12 p typically carries pluripotential genes including Nanog, Stellar, Oct4, Growth Differentiation Factor 3 (GDF3), and Polyhomeotic Homolog 1 (PHC1, Edr1) [4] and kit-ligand, KRAS proto-oncogene, GTPase (KRAS), cyclin-D, sprouty 4 (SPRY4), double sex and mab-3-related transcription factor 1 (DMRT1) and activating transcription factor 7—interacting protein (ATF-71P) which interacts with TERT expression (Tert is the catalytic component of telomerase and is upregulated in normal testis and many cancers), Bcl2-antagonist/killer 1 (BAK1) [5]; genes operating in the cell cycle including KRAS and Cyclin D2 which provide a proliferative advantage; metabolic genes driving glucose metabolism in a low oxygen environment such as Solute Carrier Family Member 3 (SLC2A3, Glut3), Glutaraldehyde-3-Phosphate Dehydrogenase (Gapdh) and Protein Tyrosine Phosphatase Non-Receptor Type 11 (PTPN11, Tp11); and genes which suppress apoptosis such as EPH Receptor A7 (EPHA7, Ek11), SRY-Box Transcription Factor 5 (Sox5) and Defender Against Cell Death 1 Pseudogene 1 (DAD1P1, Dad-R) [4]. Together these genes confer survival advantages and self-renewal, independence from supporting cell signals and apoptosis resistance and allow tumour cells to proliferate in an appropriate niche [4].

TGCT’s have usually lost parts of the Y-chromosome and chromosomes 1 p, 11, 13 and 18 and gained X, 7, 8, 12 and 21 chromosomes [4,5]. Classical oncogenes Wnt and Myc are also amplified in NSGCT [8]. There is also increasing recent concern on the role which micro-RNA’s such as the lin-28 family play in testicular oncogenesis [50]. Proprotein convertases are also implicated [51].

### 4.4. Epigenomics

Epigenomically the DNA of seminomas is completely unmethylated [4,8]. From seminomas there is a DNA methylation gradient through NSGCT. DNA methylation is low for embryonal tumours, and higher for yolk sac and teratoma tumours [8]. NSGCT are reprogrammed back to embryonal stem cells including by demethylation [4]. Embryonal stem cells express Oct4, Sox2, Nanog and Lin 28 [4]. Moreover, the genome and epigenome of embryonic stem cells is very open and very hypomethylated making it particularly vulnerable to insults of this type [4,22]. The zygote undergoes rapid DNA demethylation shortly after fertilization and most DNA methylation derived from each parent is removed. The chromatin of gonocytes, primordial germ cells and spermatogonia also has a more open configuration so it is not protected by dense heterochromatin regions with accompanying silencing polycomb protein complexes and heterochromatin as occurs later in life.

Mutations in genes controlling microtubules are also described [1].

### 4.5. Pathophysiology of Cannabinoids

Cannabis and cannabinoids are known to impact most of the above-described pathways. Cannabis is well described as inducing hypomethylation of human and rat sperm to a large degree [52,53]. In rats this effect occurs after just 12 days exposure. Moreover, this genotoxic activity including single- and double- stranded DNA breaks, micronucleus development, oxidation of all DNA nucleotides nuclear blebbing and nuclear chromosomal bridging has been described with low dose micromolar exposure to cannabidiol and its propyl analogue cannabidivarin, so that more than just the psychoactive tetrahydrocannabinol (THC) are implicated [54]. The effect of cannabis to disrupt chromosomal separation at anaphase has long been demonstrated in both lymphocytes and oocytes and dramatic photomicrographs have been published of chromosomal bridges and nuclear blebs [55], as have photomicrographs of cannabis-induced ring and chain chromosomes and micronucleus formation in sperm [56].

Moreover, in the USA, prenatal cannabis exposure has been associated across both space and time with early termination of pregnancy for anomaly (ETOPFA)- corrected rates of major chromosomal disruptions including the trisomies 21, (Downs syndrome), trisomy 18, trisomy 13, Deletion 22q11.2 and Turners syndrome [57]. Similarly Downs syndrome has been reported to have increased in relation to increased cannabis use in Canada, Colorado, Hawaii and Australia [58,59,60,61]. Prenatal cannabis use has also been linked with acute lymphoid leukaemia (ALL) development in exposed offspring (unpublished data) which is essentially a disease characterized by a variety of chromosomal translocations. In fact, if one reviews this list one finds that a variety of chromosomal derangements are noted including:

Trisomies (21, 18 and 13);
Monosomy (Turners syndrome);
Deletions (Deletion 22q11.2 and testicular cancer);
Whole genome duplications (testicular cancer);
Translocations (ALL and testicular cancer).

It therefore appears that there is an impressive array of evidence linking cannabinoid exposure to major chromosomal disruptions and rearrangements in humans.

### 4.6. Mitochondrial Bioenergetics

Moreover, cannabinoids are known to disrupt mitochondrial metabolism via several pathways [62,63,64,65,66,67,68] and hence necessarily disrupt both the epigenomic machinery and reactions involving DNA most of which are energy dependent.

From the above comments it is clear that cannabinoids induce severe morphological and functional toxicity on multiple aspects of sperm physiology and spermatogenesis especially at higher doses in the micromolar range. It is equally clear that cells exposed to cannabinoids experience genomic stress from many sources. The fact that they survive to produce pathologies, such as major congenital defects and tumourigensis, implies that cells harbouring such major genomic pathology necessarily have defective quality control mechanisms—or at least that the quality control mechanisms operating in cannabinoid-exposed cells proceed under different rules to cells which are not so exposed.

### 4.7. Endocrine Disruption

Cannabis has long been known to be an endocrine disruptor [69,70,71,72] and to be linked with both impaired testosterone production in high dose and high frequency users and impaired fertility [73,74,75,76]. There is increasing recent concern on the activity of endocrine disruption and cancer of the male germ cell line [77].

Review of this list indicates that cannabinoids are linked with:

Epigenomic DNA hypomethylation;
Chromosomal mis-segregation;

Other Chromosomal rearrangements including deletions, truncations, trisomies, monosomies, and ring and chain chromosome formation:

Micronucleus formation;
Endocrine disruption;
Microtubular damage and tubulin inhibition [62,78];
Mitochondrial inhibition.

From this brief pathophysiological overview it becomes clear that in fact all of the major steps to testicular carcinogenesis are known to be replicated in the genomic, epigenomic and mitochondriopathic toxicopathology of cannabinoids which well explains the epidemiological association of cannabis use and increased TCR demonstrated by the current epidemiological analysis and prior reports [12,13,14,15]. It is important to note therefore that where TGCT develops as a result of post-natal exposure to organochlorines or cannabinoids the usual protracted time span of tumour development from foetal life to adulthood is greatly accelerated [4]. These novel mechanistic insights also explain the strong positive effects shown in Figure 4 and may inform a consideration of such dramatic standout hotspot effects such as that shown for Hawaii in the heatmap plot of Figure 10.

Even assuming cessation of cannabis use upon identification of conception, exposure to mechanisms associated with healthy germ cell development has already likely occurred. This and exposure through other factors such as passive smoking or unintentional ingestion make it difficult for a persons involved in a cannabis use environment to know if their pregnancy was cannabis exposed. Further, given the considerable time interval between germ cell damage and TC diagnosis, cannabis exposure, which may have occurred during gestation or after is difficult to establish. Accordingly it is important to develop an objective biomarker of cannabinoid exposure such as could be derived from epigenomic or glycomic data with high sensitivity and specificity as has previously been indicated [79].

### 4.8. Generalizability

Study data is widely generalizable for several reasons. First, the study uses a large registry captured cancer databases (NPCR/SEER) from a populous nation with national and individual state data. Secondly, the NSDUH/SAMHDA database on drug/cannabis use has a good response rate. Thirdly, results are consistent across cannabinoids, confirmed using a number of different regression model systems, and consistent with all four studies to have examined the association between TCRs and Cannabis. Importantly, using inverse probability weighting and with high e-Values results fulfil the quantitative criteria of a causal relationships implying that they are robust to time and situation. Furthermore, our data fulfil all of the nine qualitative and quantitative Hill criteria of causality including strength of association, consistency amongst studies, specificity, temporality, coherence with the known data, biological plausibility, dose–response curve, analogy with similar situations elsewhere and experimental confirmation [26].

### 4.9. Strengths and Limitations

The study has a number of strengths and limitations. Strengths include the use of a large population dataset and registry controlled data and a variety of advanced statistical methods including inverse probability weighting, E-Values and geospatial regression. Not only are study findings consistent with all four studies to have examined the association but also consistent with a number of mechanistic pathways linking these epidemiological findings to well described biologically plausible modes of cannabinoid action. Our cautious view on abstinence from cannabinoids in women of reproductive age and/or wishing to conceive is shared by the American College of Obstetrics and Gynaecology and the American Academy of Pediatrics [80,81,82,83]. Study limitations relate to unavailability of individual level substance exposure data, a limitation which is common to many large epidemiological studies. The present work also does not consider cannabis migration where people with adverse childhood experiences which itself predisposes to cancer development [84] use more cannabis and so move to areas where cannabis is legal, as such information was not available to the present investigators. Two of the key methods used in the present study are inverse probability weighting and E-values. It should be appreciated that both techniques have various limitations and assumptions associated with them. IPW models are subject to potential model mis-specification or imbalanced weights [85]. These issues were addressed in this report by the straight forward specification of models. E-values are not a complete substitute for a robust sensitivity or bias analysis [86]. In the present work this was addressed by the use of several different regression techniques.

### 4.10. Conclusions

Data show that cannabis exposure has a strong dose–response relationship with TCRs and that this relationship consistent with a potential causal relationship, but do not prove causality. Data also show a strong and deleterious effect of cannabis-liberal legislative paradigms. Several cannabinoids are linked with NSGCT including THC and cannabigerol in multivariable models, and cannabinol, cannabinol, cannabichromene and cannabidiol which display bivariate dose–response relationships. The inclusion of cannabidiol on this list is of particular concern given its widely touted image as being non-psychoactive and—mistakenly—“therefore safe”. It is concerning that these findings imply an impressive acceleration of the pathobiology of TC by cannabinoids by about 20 years from the usual progression from in utero life and acceleration by the hormonal surge of adolescence, to adult/teenage toxicant exposure and peak incidence in the fourth decade of life. Moreover, the major genotoxic events leading to TC including one or more whole genome doubling events, the loss of 30–70 chromosomal arms, chromosomal translocations and genome-wide DNA demethylation, are all phenocopied precisely by many cannabinoids strengthening at once both the causal nature of the relationship and the public health importance of these findings and thereby adding considerably to the list of cannabis-induced chromosomal disorders beyond those which have been described elsewhere and broadening the pathophysiological spectrum and depth of previously described chromosomal megabase-scale genotoxicity [57].

## Figures and Tables

**Table 1:** Introductory Linear Regressions.

Parameter Estimates			Model Parameters				
Term and Model	Estimate (C.I.)	*p*-Value	S.D.	R-Squared	F	dF	*p*
***lm(Rate~Time)***							
Year—Caucasian-Americans	0.08 (0.07, 0.09)	7.80 × 10^−23^	0.3452	0.8988	374	1.41	3.20 × 10^−22^
Year—African Americans	0.011 (−0.001, 0.024)	0.0945	0.2184	0.0820	3.054	1.22	0.0945
*lm(Rate~mrjmon)*							
Cannabis	0.47 (0.34, 0.59)	7.50 × 10^−13^	0.5929	0.0652	53.25	1.748	7.49 × 10^−13^
lm(Rate~Time * mrjmon)							
Cannabis	0.47 (0.34, 0.59)	7.50 × 10^−13^	0.5928	0.0652	53.25	1.748	7.50 × 10^−13^
***Additive model***							
*lm(Rate~Cigarettes + AUD + Cannabis + Analgesics + Cocaine)*							
AUD	14.82 (12.14, 17.51)	6.46 × 10^−44^	0.5336	0.2428	49.04	5.744	6.46 × 10^−44^
Cannabis	0.45 (0.32, 0.57)	7.24 × 10^−12^					
Cocaine	−14.05 (−20.36, −7.74)	1.45 × 10^−5^					
Analgesics	−10.22 (−14.69, −5.76)	8.43 × 10^−6^					
Cigarettes	−3.19 (−4.21, −2.17)	1.64 × 10^−9^					
***Quintiles***							
*lm(Rate~Quintile)*							
Quintile 5	0.17 (0.13, 0.21)	5.2 × 10^−14^	0.1927	0.1145	25.22	4.745	1.29 × 10^−19^
lm(Rate~Year * Quintile)							
Year	0.0038 (0.0007, 0.007)	0.0184	0.1922	0.1199	21.42	5.744	4.66 × 10^−20^
Year: Quintile 5	0.17 (0.13, 0.21)	4.21 × 10^−14^					
lm(Rate~Year * Quintiles_Dichotomized)							
Year	0.0038 (0.0006, 0.007)	0.0202	0.1925	0.1165	50.4	2.747	2.94 × 10^−21^
Upper Quintiles	0.17 (0.14, 0.21)	1.37 × 10^−21^					
***Substances***							
*lm(Rate~Substances)*							
Cigarettes	−3.56 (−4.57, −2.55)	1.04 × 10^−11^	0.5949	0.0587	47.74	1.748	1.04 × 10^−11^
AUD	10.61 (7.88, 13.34)	8.51 × 10^−14^	0.5911	0.0706	57.85	1.748	8.51 × 10^−14^
Cannabis	0.47 (0.34, 0.59)	7.50 × 10^−13^	0.5928	0.0652	53.25	1.748	7.49 × 10^−13^
Analgesics	−0.62 (−0.83, −0.41)	5.42 × 10^−9^	0.5998	0.0432	34.84	1.748	5.43 × 10^−9^
Cocaine	−0.46 (−6.82, 5.9)	8.87 × 10^−1^	0.6136	−0.0013	0.0202	1.748	8.87 × 10^−1^
***Cannabinoids***							
*lm(Rate~Cannabinoids)*							
THC	0.25 (0.17, 0.34)	6.75 × 10^−9^	0.5999	0.0427	34.39	1.748	6.75 × 10^−9^
Cannabigerol	0.37 (0.26, 0.48)	3.55 × 10^−11^	0.5958	0.0557	45.19	1.748	3.55 × 10^−11^
Cannabichromene	0.45 (0.32, 0.57)	1.83 × 10^−12^	0.5935	0.0630	51.38	1.748	1.83 × 10^−142^
Cannabinol	0.24 (0.16, 0.32)	1.91 × 10^−9^	0.5989	0.0458	36.97	1.748	1.91 × 10^−9^
Cannabidiol	0.16 (0.06, 0.25)	2.25 × 10^−3^	0.6098	0.0111	9.397	1.748	2.30 × 10^−3^
Cannabis x THC Potency	0.25 (0.17, 0.34)	6.75 × 10^−9^	0.6098	0.0427	34.39	1.748	6.75 × 10^−9^

Legend: lm—linear modelling; Left Hand Side—Dependent Variable; Right Hand side—List of independent covariates; ~—Separator between left and right hand sides of a model formula; +—Additive relationship between covariates; *—Interactive relationship between covariates—includes additive relationships; AUD-Alcohol Use Disorder.

**Table 2:** Mixed Effects Regressions.

Parameter Estimates			Model Parameters			
Parameter	Estimate (C.I.)	*p*-Value	S.D.	AIC	BIC	Loglik
***Cannabis Alone—Race as Random Effects***						
*lme(Testicular_Cancer_Rate~Cannabis)*						
Cannabis	0.16 (0.15, 0.18)	1.70 × 10^−75^	0.1972	−1816.237	−1790.591	912.1183
***Additive Model***						
*lme(Testicular_Cancer_Rate~Cigarettes + AUD + Cannabis + Analgesics + Cocaine)*						
AUD	4.96 (4.59, 5.32)	3.01 × 10^−146^	0.1764	−2802.042	−2750.758	1409.021
Cannabis	0.15 (0.14, 0.17)	1.14 × 10^−68^				
Cocaine	−4.23 (−5.09, −3.38)	3.72 × 10^−22^				
Analgesics	−0.16 (−0.19, −0.13)	9.53 × 10^−31^				
Cigarettes	−1.07 (−1.21, −0.93)	2.96 × 10^−50^				
***3-Way Interactive Model***						
lme(Testicular_Cancer_Rate~Cigarettes * AUD * Cannabis + Analgesics + Cocaine)						
Cigarettes: Cannabis	11.07 (9.32, 12.82)	1.03 × 10^−34^	0.1695	−3172.38	−3095.465	1598.19
Cigarettes	27.64 (22.84, 32.44)	3.84 × 10^−29^				
AUD	64.33 (48.44, 80.22)	2.64 × 10^−15^				
Cannabis: AUD	23.66 (17.76, 29.55)	4.40 × 10^−15^				
Cocaine	−2.75 (−3.6, −1.89)	3.43 × 10^−10^				
Cigarettes: AUD	−293.19 (−361.6, −224.77)	5.95 × 10^−17^				
Cigarettes: Cannabis: AUD	−115.24 (−140.38, −90.09)	3.82 × 10^−19^				
Cannabis	−2.14 (−2.54, −1.74)	1.57 × 10^−25^				
Analgesics	−0.15 (−0.18, −0.12)	3.76 × 10^−29^				
***4-Way Interactive Model***						
lme(Testicular_Cancer_Rate~Cigarettes * AUD * Cannabis * Analgesics + Cocaine)						
Cannabis: Analgesics	2.08 (1.64, 2.51)	9.32 × 10^−21^	0.1684	−3212.364	−3116.23	1621.182
Analgesics	5.44 (4.3, 6.58)	1.12 × 10^−20^				
Cigarettes: Cannabis: AUD: Analgesics	33.83 (25.81, 41.85)	1.78 × 10^−16^				
Cigarettes: AUD: Analgesics	85.86 (63.96, 107.76)	1.87 × 10^−14^				
Cannabis	4.4 (3.09, 5.71)	4.51 × 10^−11^				
Cocaine	−1.94 (−2.8, −1.07)	1.15 × 10^−05^				
Cigarettes: Cannabis	−20.18 (−26.02, −14.34)	1.41 × 10^−11^				
Cannabis: AUD: Analgesics	−6.68 (−8.56, −4.79)	4.30 × 10^−12^				
Cigarettes	−55.44 (−71.02, −39.86)	3.54 × 10^−12^				
AUD: Analgesics	−18.32 (−23.42, −13.22)	2.14 × 10^−12^				
Cigarettes: Analgesics	−26.55 (−31.79, −21.31)	4.98 × 10^−23^				
Cigarettes: Cannabis: Analgesics	−9.99 (−11.95, −8.02)	3.75 × 10^−23^				
***4-Way Interactive Model with Income***						
lme(Testicular_Cancer_Rate~Cigarettes * AUD * Cannabis * Analgesics + Cocaine + Income)						
Analgesics	5.46 (4.33, 6.59)	5.04 × 10^−21^	0.1674	−3258.286	−3155.747	1645.143
Cannabis: Analgesics	2.04 (1.61, 2.47)	3.17 × 10^−20^				
Cigarettes: Cannabis: AUD: Analgesics	34.58 (26.61, 42.56)	2.57 × 10^−17^				
Cigarettes: AUD: Analgesics	87.71 (65.94, 109.49)	3.63 × 10^−15^				
log(MHY)	0.16 (0.12, 0.21)	2.38 × 10^−13^				
Cannabis	4.25 (2.95, 5.54)	1.70 × 10^−10^				
Cocaine	−2.15 (−3.01, −1.29)	1.08 × 10^−6^				
Cigarettes: Cannabis	−20.22 (−26.03, −14.42)	9.74 × 10^−12^				
Cigarettes	−56.6 (−72.1, −41.11)	9.34 × 10^−13^				
Cannabis: AUD: Analgesics	−6.91 (−8.78, −5.04)	5.83 × 10^−13^				
AUD: Analgesics	−18.86 (−23.93, −13.79)	3.56 × 10^−13^				
Cigarettes: Cannabis: Analgesics	−10 (−11.95, −8.04)	1.96 × 10^−23^				
Cigarettes: Analgesics	−27.04 (−32.25, −21.83)	4.60 × 10^−24^				

Legend: See Table 1. lme—Mixed effects regressions; A.I.C.—Akaike Information Criterion; B.I.C.—Bayesian Information Criterion; logLik—Log of the Maximum Likelihood Ratio at model optimization; *—interaction term between covariates.

**Table 3:** Robust Inverse Probability Weighted Regressions.

Parameter	Estimate (C.I.)	*p*-Value
***Additive Model with State Cannabis***		
*svyglm(TestCaRt~Cigarettes + Cannabis + Race + AUD + Analgesics + Cocaine)*		
AUD	64.55 (56.89, 72.22)	<2.2 × 10^−16^
Analgesics	11.01 (6.24, 15.77)	5.7 × 10^−5^
NHWhite	5.44 (2.36, 8.53)	0.0013
Hispanic	4.63 (1.54, 7.71)	0.0055
Cannabis	−9.38 (−12.76, −5.99)	3.4 × 10^−6^
Cocaine	−131.14 (−138.96, −123.33)	<2.2 × 10^−16^
***Additive Model with Ethnic THC Exposure***		
svyglm(TestCaRt~Cigarettes * EthnicTHCExposure * Race + AUD + Analgesics + Cocaine)		
Cigarettes	28.96 (27.87, 30.05)	<2 × 10^−16^
Hispanic	2.55 (2.05, 3.04)	1.8 × 10^−12^
Asian	4.89 (1.08, 8.69)	0.0160
EthnicTHCExposure	2.9 (0.41, 5.38)	0.0281
Cocaine	−56.03 (−110.14, −1.93)	0.0492
***Interactive Model with Ethnic_THC_Exposure***		
svyglm(TestCaRt~Cigarettes * EthnicTHCExposure * Race + AUD + Analgesics + Cocaine + Income)		
EthnicTHCExposure	4.72 (2.04, 7.41)	0.0018
Asian	3.93 (1.64, 6.22)	0.0022
Cigarettes	11.09 (3.77, 18.42)	0.0060
Cigarettes: NHWhite	109.87 (24.24, 195.5)	0.0177
Cigarettes: EthnicTHCExposure: Asian	17.32 (1.64, 33.01)	0.0388
NHWhite	−19.53 (−39.11, 0.05)	0.0603
EthnicTHCExposure: Asian	−4.45 (−8.41, −0.5)	0.0352
Cocaine	−88.11 (−161.51, −14.7)	0.0257
Cigarettes: EthnicTHCExposure	−20.11 (−32.44, −7.78)	0.0034
Cigarettes: Asian	−16.35 (−26.11, −6.6)	0.0027
***Interactive Model with State Cannabis***		
svyglm(TestCaRt~Cigarettes * Cannabis * Race + AUD + Analgesics + Cocaine + Income)		
AUD	13.58 (7.69, 19.47)	0.0001
Cigarettes: Cannabis: NHWhite	398.68 (214.58, 582.79)	0.0002
Cigarettes: NHWhite	933.24 (493.05, 1373.44)	0.0003
Cigarettes: Hispanic	1372.45 (672.78, 2072.11)	0.0007
Cigarettes: Cannabis: Hispanic	635.8 (305.36, 966.24)	0.0008
Analgesics	19.88 (9.19, 30.57)	0.0011
Cannabis	42.63 (18.65, 66.61)	0.0017
Cigarettes: Cannabis	−199.68 (−315.91, −83.44)	0.0023
Cigarettes	−578.67 (−907.11, −250.23)	0.0018
Cocaine	−75.51 (−117.23, −33.78)	0.0015
Cannabis: Hispanic	−144.06 (−218.53, −69.59)	0.0008
Hispanic	−308.36 (−466.63, −150.09)	0.0007
NHWhite	−214 (−315.14, −112.87)	0.0003
Cannabis: NHWhite	−93.06 (−135.37, −50.75)	0.0002
***Interactive Model with State Cannabinoids***		
svyglm(TestCaRt~Cigarettes * THC * Cannabigerol * Race + AUD + Analgesics + Cocaine + Income)		
Cigarettes: NHWhite	127.24 (92.37, 162.1)	4.7 × 10^−7^
Cigarettes: THC: Cannabigerol: NHBlack	13.87 (6.33, 21.41)	0.0017
Cigarettes: NHBlack	53.77 (18.14, 89.41)	0.0075
Cigarettes: THC: NHBlack	49.95 (14.93, 84.96)	0.0108
Cigarettes: Cannabigerol: NHBlack	14.62 (3.67, 25.57)	0.0161
Cigarettes: THC: Cannabigerol: NHWhite	35.97 (7.97, 63.97)	0.0200
Cigarettes: THC: Hispanic	214.68 (31.83, 397.53)	0.0317
Cigarettes: Hispanic	−310.38 (−540.45, −80.31)	0.0152
Cigarettes: Cannabigerol: Hispanic	−89.37 (−155.46, −23.28)	0.0150
AUD	−43.07 (−68.76, −17.38)	0.0035
THC: Cannabigerol: NHWhite	−8.18 (−11.39, −4.96)	6.2 × 10^−5^
NHWhite	−16.98 (−23.16, −10.81)	2.4 × 10^−5^

Legend: See Table 1. Svyglm—Robust generalized linear regression; TestCaRt—Testicular Cancer Rate; *—interaction term between covariates.

**Table 4:** Geospatiotemporal Regressions.

Lagged Variables	Parameter			Model				
	Parameter	Estimate (C.I.)	*p*-Value	LogLik	S.D.	Model Parameter	Estimate	*p*-Value
	*spreml(Rate~Cannabis)*					phi	1.2910	1.4 × 10^−5^
	Cannabis	0.19 (0.1, 0.28)	3.4 × 10^−5^	−390.8963	0.3939	psi	−0.1114	0.0055
						rho	−0.3795	0.0055
						lambda	0.4298	2.1 × 10^−5^
	*spreml(Rate~Cigarettes + AUD + Cannabis + Analgesics + Cocaine)*					phi	1.2910	1.4 × 10^−5^
	Cannabis	0.19 (0.1, 0.28)	3.4 × 10^−5^	−390.8693	0.5316	psi	−0.1114	0.0055
						rho	−0.3795	0.0055
						lambda	0.4298	2.1 × 10^−5^
	spreml(Rate~Cigarettes * Cannabis * AUD + Analgesics + Cocaine)					phi	1.2650	2.6 × 10^−5^
	Cigarettes: Cannabis	0.36 (0.19, 0.53)	4.6 × 10^−5^	−391.1668	0.5300	psi	−0.1101	0.0062
						rho	−0.3696	0.0088
						lambda	0.4242	5.2 × 10^−5^
	spreml(Rate~Cigarettes * Cannabis * AUD + Analgesics + Cocaine + Income + 5_Races)							
	CaucAsian-Am.	1.59 (1.26, 1.93)	<2.2 × 10^−16^	−353.3539	0.1584	phi	0.1846	0.0004
	Hispanic-Am.	0.1 (0.03, 0.16)	6.0 × 10^−3^			psi	−0.0891	0.0284
	Asian-Am.	0.13 (0.07, 0.2)	8.3 × 10^−5^			rho	−0.1766	0.2595
	African-Am.	−0.23 (−0.27, −0.19)	<2.2 × 10^−16^			lambda	0.2183	0.0847
	spreml(Rate~Cigarettes * THC * Cannabigerol * AUD + Analgesics + Cocaine + Income + 5_Races)							
	CaucAsian-Am.	1.6 (1.16, 2.03)	8.0 × 10^−13^	−348.3428	0.3929	phi	0.1572	0.0009
	Hispanic-Am.	0.11 (0.04, 0.18)	0.0028			psi	−0.0955	0.0197
	Asian-Am.	0.11 (0.03, 0.19)	0.0074			rho	−0.1642	0.1982
	Cigarettes: Cannabigerol	1.39 (0.24, 2.53)	0.0177			lambda	0.1945	0.0531
	THC: Cannabigerol	0.07 (0.01, 0.12)	0.0187					
	Cigarettes	4.45 (0.34, 8.56)	0.0340					
	Analgesics	−0.18 (−0.36, 0)	0.0457					
	African-Am.	−0.22 (−0.26, −0.17)	<2.2 × 10^−16^					
	***2 Spatial Lags***							
	spreml(Rate~Cigarettes * THC * Cannabigerol * AUD + Analgesics + Cocaine + Income + 5_Races)							
CBG, 2	Caucasian-Am.	1.63 (1.18, 2.08)	1.2 × 10^−12^	−351.162	0.3970	phi	0.1882	0.0004
	Asian-Am.	0.14 (0.06, 0.22)	0.0007			psi	−0.0976	0.0167
	Hispanic-Am.	0.1 (0.03, 0.17)	0.0054			rho	−0.1825	0.2500
	Cigarettes: THC: Cannabigerol	0.71 (0.05, 1.37)	0.0350			lambda	0.2199	0.0845
	Cigarettes: THC	2.58 (0.17, 4.98)	0.0356					
	African-Am.	−0.23 (−0.27, −0.19)	<2.2 × 10^−16^					
	***2 Temporal Lags***							
	spreml(Rate~Cigarettes * THC * Cannabigerol * AUD + Analgesics + Cocaine + Income + 5_Races)							
CBG, 2	Caucasian-Am.	1.8 (1.33, 2.26)	4.6 × 10^−14^	−294.7663	0.3863	phi	0.1487	0.0012
	Asian-Am.	0.16 (0.08, 0.23)	5.2 × 10^−5^			psi	−0.0972	0.0289
	Hispanic-Am.	0.15 (0.07, 0.22)	7.3 × 10^−5^			rho	−0.1770	0.2840
	Cigarettes: THC	23.6 (11.92, 35.29)	7.5 × 10^−5^			lambda	0.2025	0.1205
	Cigarettes: THC: Cannabigerol	6.22 (3.07, 9.37)	0.0001					
	Cannabigerol	0.18 (0.04, 0.32)	0.0146					
	Analgesics	−0.27 (−0.44, −0.09)	0.0031					
	THC: Cannabigerol	−1.26 (−1.94, −0.57)	0.0003					
	THC	−4.86 (−7.36, −2.37)	0.0001					
	African-Am.	−0.22 (−0.26, −0.18)	<2.2 × 10^−16^					
	***Full Model with Ethnic THC Exposure***							
	spreml(Rate~Cigarettes * AUD + Analgesics + Cocaine + MHY + NHCaucasian-Am._THC_Exposure * NHAfrican-Am._THC_Exposure * Hispanic-Am._THC_Exposure + Asian-Am._THC_Exposure + AIAN_THC_Exposure)							
	NHAfrican-Am._THC_Exposure	0.15 (0.06, 0.25)	0.0009	−380.0512	0.4856	phi	1.0087	1.1 × 10^−5^
	Asian-Am._THC_Exposure	−0.1 (−0.19, −0.02)	0.0173			psi	−0.1142	0.0044
						rho	−0.5161	3.9 × 10^−8^
						lambda	0.4720	4.4 × 10^−13^

Legend: See Table 1. Spreml—Spatial Panel Random Effects Maximum Likelihood Regression; 5_Races—Caucasian- African- Hispanic- Asian- American Indian/Alaskan Native- American ancestry; phi—Random error coefficient; psi—Serial correlation coefficient; rho—Spatial error coefficient; lambda—Spatial error autocorrelation coefficient; * interaction term between covariates.

**Table 5:** Selected e-Values.

Parameter	Estimate (C.I.)	R.R. (C.I.)	E-Values
*LINEAR MODELS*			
***Testicular_Cancer~Cannabis***			
Cannabis	0.47 (0.34, 0.59)	2.04 (1.69, 2.47)	3.50, 2.76
Testicular_Cancer~Time * Cannabis			
Cannabis	0.47 (0.34, 0.59)	2.04 (1.68, 2.47)	3.50, 2.76
*Additive Drug Model*			
Cannabis	0.45 (0.32, 0.57)	2.14 (1.73, 2.65)	3.70, 2.85
*Testicular_Cancer~Cannabis Quintiles*			
Quintile 5	0.17 (0.13, 0.21)	2.13 (1.75, 2.58)	3.68, 2.91
Testicular_Cancer~Time * Cannabis_Quintiles			
Year: Quintile 5	0.17 (0.13, 0.21)	2.23 (1.82, 2.73)	3.88, 3.04
Testicular_Cancer~Time * Dichotomized_Cannabis_Quintiles			
Upper Quintiles	0.17 (0.14, 0.21)	2.24 (1.91, 2.64)	3.91, 3.22
*Substances*			
Cannabis	0.47 (0.34, 0.59)	2.04 (122.69, 2.43)	3.50, 2.76
THC	0.25 (0.17, 0.34)	1.47 (1.29, 1.67)	2.30, 1.91
Cannabigerol	0.37 (0.26, 0.48)	1.76 (1.49, 2.08)	2.92, 2.35
Cannabichromene	0.45 (0.32, 0.57)	1.98 (1.64, 2.39)	3.38, 2.68
Cannabinol	0.24 (0.16, 0.32)	1.43 (1.27, 1.61)	2.23, 1.88
Cannabidiol	0.16 (0.06, 0.25)	1.26 (1.08, 1.46)	1.83, 1.40
Cannabis x THC Potency	0.25 (0.17, 0.34)	1.46 (1.28, 1.66)	2.28, 1.90
***MIXED EFFECTS***			
*Cannabis Alone—Race as Random Effects*			
Cannabis	0.16 (0.15, 0.18)	3.51 (3.08, 4.00)	6.48, 5.61
***Additive Model***			
Cannabis	0.15 (0.14, 0.17)	3.29 (2.89, 3.76)	6.05, 5.23
***3-Way Interactive Model***			
Cigarettes: Cannabis	11.07 (9.32, 12.82)	9.67 × 10^25^ (7.66 × 10^21^, 1.22 × 10^30^)	1.95 × 10^26^, 1.54 × 10^22^
Cannabis: AUD	23.66 (17.76, 29.55)	3.23 × 10^55^ (5.17 × 10^41^, 2.02 × 10^69^)	6.45 × 10^55^, 1.03 × 10^42^
***4-Way Interactive Model***			
Cannabis: Analgesics	2.08 (1.64, 2.51)	7.48 × 10^4^ (7.22 × 10^3^, 7.75 × 10^5^)	1.49 × 10^5^, 1.44 × 10^4^
Cigarettes: Cannabis: AUD: Analgesics	33.83 (25.81, 41.85)	2.41 × 10^79^ (4.00 × 10^60^, 1.46 × 10^98^)	4.84 × 10^79^, 8.01 × 10^60^
Cannabis	4.4 (3.09, 5.71)	2.13 × 10^10^ (1.85 × 10^8^, 2.45 × 10^13^)	4.26 × 10^10^, 3.71 × 10^8^
***4-Way Interactive Model with Income***			
Cannabis: Analgesics	2.04 (1.61, 2.47)	6.41 × 10^4^ (6.18 × 10^3^, 6.64 × 10^5^)	1.28 × 10^5^, 1.24 × 10^4^
Cigarettes: Cannabis: AUD: Analgesics	34.58 (26.61, 42.56)	4.31 × 10^81^ (7.03 × 10^62^, 2.64 × 10^100^)	8.61 × 10^81^, 1.40 × 10^63^
Cannabis	4.25 (2.95, 5.54)	1.05 × 10^10^ (9.08 × 10^6^, 1.21 × 10^13^)	2.09 × 10^10^, 1.82 × 10^7^
***GEOSPATIAL MODELS***			
*spreml(Rate~Cannabis)*			
Cannabis	0.19 (0.1, 0.28)	1.55 (1.18, 2.05)	2.48, 1.64
*spreml(Rate~Cigarettes + AUD + Cannabis + Analgesics + Cocaine)*			
Cannabis	0.19 (0.1, 0.28)	1.39 (1.19, 1.62)	2.12, 1.66
spreml(Rate~Cigarettes * Cannabis * AUD + Analgesics + Cocaine)			
Cigarettes: Cannabis	0.36 (0.19, 0.53)	1.85 (1.38, 2.49)	3.11, 2.10
spreml(Rate~Cigarettes * THC * Cannabigerol * AUD + Analgesics + Cocaine + Income + 5_Races)			
Cigarettes: Cannabigerol	1.39 (0.24, 2.53)	24.77 (1.76, 349.61)	49.05, 2.91
THC: Cannabigerol	0.07 (0.01, 0.12)	1.16 (1.03, 1.32)	1.60, 1.19
***2 Spatial Lags***			
Cigarettes: THC: Cannabigerol	0.71 (0.05, 1.37)	5.09 (1.12, 23.10)	9.66, 1.50
Cigarettes: THC	2.58 (0.17, 4.98)	368.56 (1.51, 9.02 × 10^4^)	736.63, 2.38
***2 Temporal Lags***			
Cigarettes: THC	23.6 (11.92, 35.29)	1.40 × 10^24^ (1.64 × 10^12^, 1.20 × 10^36^)	2.81 × 10^24^, 3.29 × 10^12^
Cigarettes: THC: Cannabigerol	6.22 (3.07, 9.37)	2.31 × 10^6^ (1.39 × 10^3^, 3.82 × 10^9^)	4.62 × 10^6^, 2.79 × 10^3^
Cannabigerol	0.18 (0.04, 0.32)	1.52 (1.09, 2.13)	2.41, 1.39
spreml(Rate~Cigarettes * AUD + Analgesics + Cocaine + MHY + NHCaucasian-Am._THC_Exposure * NHAfrican-Am._THC_Exposure * Hispanic-Am._THC_Exposure + Asian-Am._THC_Exposure + AIAN_THC_Exposure)			
NHAfrican-Am._THC_Exposure	0.15 (0.06, 0.25)	1.33 (1.13, 1.58)	2.00, 1.51
***LEGAL STATUS***			
Legal	0.14 (0.06, 0.22)	1.88, (1.33, 2.66)	3.16, 1.98
Medical	0.05 (0.01, 0.09)	1.25 (1.06, 1.48)	1.82, 1.31
***Legal Status Over Time***			
Legal	0.11 (0.03, 0.19)	1.63 (1.12, 2.34)	2.63, 1.50
***Dichotomized Status***			
Liberal	0.05 (0.02, 0.08)	1.25 (1.10, 1.43)	1.81, 1.43
***Dichotomized Status Over Time***			
Liberal	0.05 (0.02, 0.08)	1.25 (1.10, 1.43)	1.81, 1.43
***Quintiles***			
Quintile 5	0.20 (0.13, 0.21)	2.13 (1.75, 2.58)	3.68, 2.91
***Dichotomized Quintiles***			
Upper_2_Quintiles	0.20 (0.06, 0.35)	2.24 (1.91, 2.64)	3.91, 3.22

Abbreviations: R.R.—Relative Risk. * interaction term between covariates.

**Table 6:** Ordered e-Value Lists.

No.	E-Value Estimates	Minimum E-Value
1	8.61 × 10^81^	1.40 × 10^63^
2	4.84 × 10^79^	8.01 × 10^60^
3	6.45 × 10^55^	1.03 × 10^42^
4	1.95 × 10^26^	1.54 × 10^22^
5	2.81 × 10^24^	3.29 × 10^12^
6	4.26 × 10^10^	3.71 × 10^8^
7	2.09 × 10^10^	1.82 × 10^7^
8	4.62 × 10^6^	14,400.00
9	1.49 × 10^5^	12,400.00
10	1.28 × 10^5^	2790.00
11	736.63	5.61
12	49.05	5.23
13	9.66	3.22
14	6.48	3.22
15	6.05	3.04
16	3.91	2.91
17	3.91	2.91
18	3.88	2.91
19	3.70	2.85
20	3.68	2.76
21	3.68	2.76
22	3.50	2.76
23	3.50	2.68
24	3.50	2.38
25	3.38	2.35
26	3.16	2.10
27	3.11	1.98
28	2.92	1.91
29	2.63	1.9
30	2.48	1.88
31	2.41	1.66
32	2.30	1.64
33	2.28	1.51
34	2.23	1.50
35	2.12	1.50
36	2.00	1.43
37	1.83	1.43
38	1.82	1.40
39	1.81	1.39
40	1.81	1.31
41	1.60	1.19

**Table 7:** Effect of Cannabis Legalization.

Linear Models							
Parameter Estimates			Model Parameters				
Parameter	Estimate (C.I.)	*p*-Value	S.D.	R-Squared	F	dF	*p*
***Legal Status***							
*(lm(Rate~Status)*							
Decriminalized	0.03 (−0.01, 0.07)	0.0979	0.2029	0.0196	5.979	3.746	0.0005
Legal	0.14 (0.06, 0.22)	0.0004					
Medical	0.05 (0.01, 0.09)	0.0086					
(lm(Rate~Year * Status)							
Legal	0.11 (0.03, 0.19)	0.0102	0.2029	1.96 × 10^−2^	5.979	3.746	0.0005
*(lm(Rate~Dichotomized Status)*							
Liberal	0.05 (0.02, 0.08)	0.0008	0.2035	0.0135	11.24	1.748	0.0008
(lm(Rate~Year * Dichotomized Status)							
Liberal	0.05 (0.02, 0.08)	0.0008	0.2035	0.0135	11.24	1.748	0.0008
**Linear Models from Imputed Dataset**							
**Parameters**			**Model**				
**Parameter**	**Estimate (C.I.)**	***p*-Value**	**No. Imputations**		**SD**	**lambda**	**FMI**
***From Imputed Dataset***							
***Dichotomized Quintiles***							
*lm(TestCaRt~Dichotomized_Quintiles)*							
Upper_2_Quintiles	0.20 (0.06, 0.35)	0.0058	256		1.8375	0.0309	0.0316

Abbreviations: FMI—Fraction of missing information; Lambda—Proportion of information which is due to missing data; * interaction term.

## Data Availability

All data generated or analysed during this study are included in this published article and its Supplementary Materials files. Data along with the relevant R code has been made publicly available on the Mendeley Database Repository and can be accessed from these URL’s: http://doi.org/10.17632/ttzb9xvb4v.1 (accessed on 18 October 2020). All authors had full access to all the data in the study and take responsibility for the integrity of the data and the accuracy of the data analysis.

## Supplementary Materials

The following supporting information can be downloaded at: https://www.mdpi.com/article/10.3390/ijerph191912759/s1, Table S1: Testicular Cancer Rates USA by State and Year 2001–2017; Table S2: States in Each Cannabis Use Quintile; Figure S1: Map of states with available data. (A) Last month cannabis use and (B) testicular cancer incidence rates across USA by state for 2017.
