# Supplementary material for: State Trends of Cannabis Liberalization as a Causal Driver of Increasing Testicular Cancer Rates across the USA
Source: Int J Environ Res Public Health. 2022 Oct 5;19(19):12759. doi: 10.3390/ijerph191912759 (PMC9565972; doi:10.3390/ijerph191912759)
Supplement: Supplementary file 1 [file ijerph-19-12759-s001.zip › ijerph-1875316-supplementary.pdf]

Supplementary Table S1.: Testicular Cancer Rates USA by State and Year 2001 – 2017.

**State**	**Year**																
	**2001**	**2002**	**2003**	**2004**	**2005**	**2006**	**2007**	**2008**	**2009**	**2010**	**2011**	**2012**	**2013**	**2014**	**2015**	**2016**	**2017**
Alaska	3.9	4.3	3.3	3	3.5	3.2	2.4	3.2	2.8	3.9	3.5	3	3.1	4.1	4.3	3.1	2.7
Alabama	2	2.2	1.9	2.4	2.4	2	2.3	2.7	2.4	2.5	2	2.1	2.2	2.6	2.1	2	2.1
Arkansas	2.2	1.8	2.8	2.1	2.3	1.9	2.3	2.1	2.5	2.4	2.1	2.6	2.5	3.1	2.3	2.9	2.7
Arizona	2.6	2.7	2.8	2.8	2.9	3.2	2.7	3	3.1	2.8	3	2.9	2.4	2.8	2.9	2.8	2.8
California	2.7	2.6	2.7	2.8	2.8	2.9	2.9	3	3	3.1	2.9	3	3.1	3.2	3.1	3.2	3.2
Colorado	3	3.1	3.4	3.2	3.1	2.9	3.2	3.3	3.2	3.3	2.8	3	2.9	3.1	3.2	2.8	3
Connecticut	2.8	3.3	3	3.5	3	3.2	3.7	3	3.4	2.9	3.3	3.4	3.5	3.7	2.4	3.7	3.3
Delaware	2.9	2	2.5	**2.65**	2.8	2.5	2.8	2.5	3	3	2	2.9	3.8	2.6	2.6	2.5	1.8
Florida	2.8	2.6	2.8	2.5	2.8	2.9	2.6	2.7	2.6	2.4	2.4	2.7	2.6	2.4	2.4	2.7	2.4
Georgia	2.2	2.2	2.2	2.1	1.9	2.1	2.1	2.3	2.1	2	2.1	2	2.1	2.2	2.1	2.4	2.2
Hawaii	2.5	2.1	2.3	2.3	3.3	2.6	2.9	3.4	3.5	2.6	2.3	2.8	2.7	3.3	3.3	3.4	2.7
Idaho	2.9	3	3.5	4.3	3.9	3.2	3.1	3.4	3.4	2.9	4.5	3	2.9	4.3	4	3.9	3
Illinois	2.7	2.8	2.7	2.8	2.7	2.9	2.7	3	2.9	3.1	2.8	3.1	3.1	3	3.3	3.4	3.1
Indiana	3.1	2.6	3.2	2.8	2.5	2.7	3	2.8	2.7	2.9	2.9	2.6	3.3	3.2	3.4	3.2	2.5
Iowa	3.9	3.3	3.3	3.5	3.8	3.3	3.3	3.4	3.5	3.9	3.9	3.2	3.5	3.6	4.1	3.6	4
Kansas	2.6	2.3	2.7	2.8	3.4	2.9	3.1	3.2	3.5	2.9	2.8	3.9	2.9	3.7	3.5	3.1	3.1
Kentucky	2.8	2.8	2.3	2.4	2.8	2.7	2.3	3	2.7	3.1	3	2.6	2.9	3	2.8	3	3
Louisiana	2.4	2.2	2.3	2.6	2.2	2.1	2.2	2.2	2.5	2.3	2	2.7	2.3	3	2.2	2.3	2.4
Massachusetts	3.1	3.5	3.2	3	3.6	3.2	3.4	3.1	3.5	2.8	3.4	3.4	3.4	3.3	3.1	2.8	2.7
Maryland	2.2	2.6	2.5	1.9	2.2	2.1	2.3	2.3	2.2	2.1	2.3	2.6	2.4	2.3	2.4	2.3	2.3
Maine	3.1	3.2	3.6	3.1	3.8	3.1	3.1	3.5	3.1	4.3	3.5	3.2	3.8	3	3.6	3.8	4.2
Michigan	3	2.9	2.9	3.2	3.2	2.9	3.1	2.8	2.6	3.2	3	3	3.3	3	3	2.9	3.1
Minnesota	3.8	3.8	3.8	3.5	4	3	3.9	3.3	4	3.4	3.6	3.7	3.7	3.4	3.7	3.5	3.5
Missouri	3	2.6	2.5	2.7	2.8	2.6	2.2	2.7	2.7	2.8	2.5	2.6	3	2.4	3.2	2.8	2.8

Mississippi	**1.7**	**1.5**	1.3	2.2	2	1.8	2	1.9	1.5	2.3	1.6	2.1	1.7	2.1	1.8	2.1	2.4
Montana	2.8	3.7	3.8	2.9	3.7	3	2.7	3.7	2.4	5.6	3.8	4.1	3.4	3.6	4.3	3.8	3.5
North Carolina	2.7	2.3	2.4	2.6	2.5	2.8	2.6	2.5	2.8	2.3	2.5	2.4	2.6	2.3	2.7	2.9	2.6
North Dakota	3.7	3.1	2.7	4	2.9	3.4	2.9	3.2	5	3.7	2.6	**3.1**	3.6	4.2	3.4	4.4	3.8
Nebraska	4	2.6	3.7	3.3	3.2	3.2	3.9	3.3	3.4	3.3	3.4	3.6	3.6	4.5	3.2	4.1	3.3
New Hampshire	3.7	3.2	4	4.1	3.1	4.3	3.8	3.8	4.1	4.1	3.8	4.3	4.7	2.4	3.7	4.1	3.9
New Jersey	3.2	3.2	3	3.2	2.6	2.9	3	2.8	3	3	3.1	3	3.2	2.9	3.1	3	3.1
New Mexico	3.2	3.5	2.8	2.6	3.5	3.3	3.1	3.5	3	3.3	3.4	2.9	3.8	3.5	3.4	3.2	3.3
Nevada	2.4	2	3.3	2.6	2.7	2.8	2.4	2.4	2.8	2.5	2.4	2.7	2.3	2.7	2.3	3.5	3.5
New York	2.6	2.5	2.8	2.8	2.9	2.8	3	3.1	2.9	3	3	3.2	2.9	3	2.9	3.1	3.1
Ohio	3	2.9	2.7	3	3.4	2.7	2.9	2.8	2.9	2.6	3	2.8	2.8	3.2	3.2	2.8	2.7
Oklahoma	2.3	2.4	3.2	2.6	2.7	2.6	2.5	2.8	2.7	2.5	3	2.3	2.8	3.1	2.6	3.2	2.3
Oregon	4.1	3.7	3.5	3.4	3.3	3.2	3.8	3.5	3.1	3.2	3.6	3.2	3.7	3.9	3.6	3.3	3.2
Pennsylvania	3.2	3.3	2.9	3.6	3.2	3.3	3	3.4	3.4	3.8	3.3	3.5	3.4	3.2	3.4	3.6	2.9
Rhode Island	3	2.9	2.4	3.8	3.4	1.9	3.7	3.9	2.8	3.2	4.1	4.1	3.6	1.9	4.3	2.8	3.1
South Carolina	2	1.8	2.4	1.8	2.1	2.3	1.9	2.3	2	2.1	2.3	2.3	2.1	2.2	2.1	2.2	2
South Dakota	3.5	3.7	3.7	2.6	4.4	2.8	2.6	3.8	2.5	3.4	2.6	3.3	4	4.4	3.2	2.5	2.4
Tennessee	2	1.9	2.1	2.3	2.9	2.4	2.5	2.6	2.3	2.4	2.6	2.5	2.9	2.8	2.5	2.9	3
Texas	2.5	2.5	2.7	2.6	2.6	2.7	2.5	2.7	2.5	2.5	2.4	2.5	2.7	2.6	2.6	2.7	2.8
Utah	2.8	4	4.1	4.4	3.1	3	4.4	3.7	3.7	3.7	3.4	3.7	3.7	3.4	3.8	3.9	4.4
Virginia	2	2.2	2.3	2.5	2.9	2.5	2.5	2.5	2.2	2.5	2.2	2	2.2	2.2	2.1	2.3	2.3
Vermont	4.8	3.1	2.8	4.8	**3.9**	3	3.6	3.5	3.9	2.8	3.8	**4.25**	4.7	3.6	**4.05**	4.5	3.8
Washington	3.4	3.3	3.2	3.3	3.6	3.8	3.2	3.2	3.8	3.4	3.5	3.7	3.4	4	3.1	3.6	3.2
Wisconsin	3.3	3.5	3.5	3.7	3.7	3.6	3.7	3.3	3.5	3.9	3.6	3.8	3.3	3.7	3.7	3.9	3.4
West Virginia	2.1	2.4	3	2.5	3	2.6	3.1	2.9	3.2	3.5	2.8	3.6	2.9	2.8	3	2.7	2.7
Wyoming	4.3	**4.23**	**4.15**	**4.08**	4	**3.7**	3.4	**3.4**	**3.4**	3.4	4.2	3	5.7	3.3	3.5	4.1	4.4

Supplementary Table S1. State based CDC age-adjusted testicular cancer rates 2001-2017 as raw data with temporally kriged data marked in bold and red typeface.

Supplementary Table S2.: States in Each Cannabis Use Quintile

State	Quintile 1	Quintile 2	Quintile 3	Quintile 4	Quintile 5
Alabama	14	1	0	0	0
Alaska	0	0	0	0	15
Arizona	0	3	7	5	0
Arkansas	0	6	9	0	0
California	0	0	0	15	0
Colorado	0	0	0	1	14
Connecticut	0	0	2	12	1
Delaware	0	0	6	9	0
Florida	0	0	14	1	0
Georgia	2	3	8	2	0
Hawaii	0	1	4	8	2
Idaho	6	9	0	0	0
Illinois	0	2	13	0	0
Indiana	0	1	2	12	0
Iowa	14	1	0	0	0
Kansas	4	11	0	0	0
Kentucky	0	7	7	1	0
Louisiana	7	8	0	0	0
Maine	0	0	0	0	15
Maryland	2	3	1	9	0
Massachusetts	0	0	0	3	12
Michigan	0	0	0	15	0
Minnesota	0	3	7	4	1
Mississippi	14	1	0	0	0
Missouri	0	0	14	1	0
Montana	0	0	0	0	15
Nebraska	9	4	2	0	0
Nevada	0	0	4	9	2
New Hampshire	0	0	0	0	15
New Jersey	8	7	0	0	0
New Mexico	0	0	0	15	0
New York	0	0	0	15	0
North Carolina	0	11	4	0	0
North Dakota	15	0	0	0	0
Ohio	0	0	13	2	0
Oklahoma	10	4	1	0	0
Oregon	0	0	0	0	15
Pennsylvania	0	2	12	1	0
Rhode Island	0	0	0	0	15
South Carolina	4	6	5	0	0

South Dakota	7	7	1	0	0
Tennessee	1	8	4	2	0
Texas	15	0	0	0	0
Utah	15	0	0	0	0
Vermont	0	0	0	0	15
Virginia	2	8	4	1	0
Washington	0	0	0	2	13
West Virginia	1	12	2	0	0
Wisconsin	0	14	1	0	0
Wyoming	6	6	2	1	0

Supplementary Table S2. State composition of each quintile.

Figure S1 - States in US Testicular Cancer Dataset

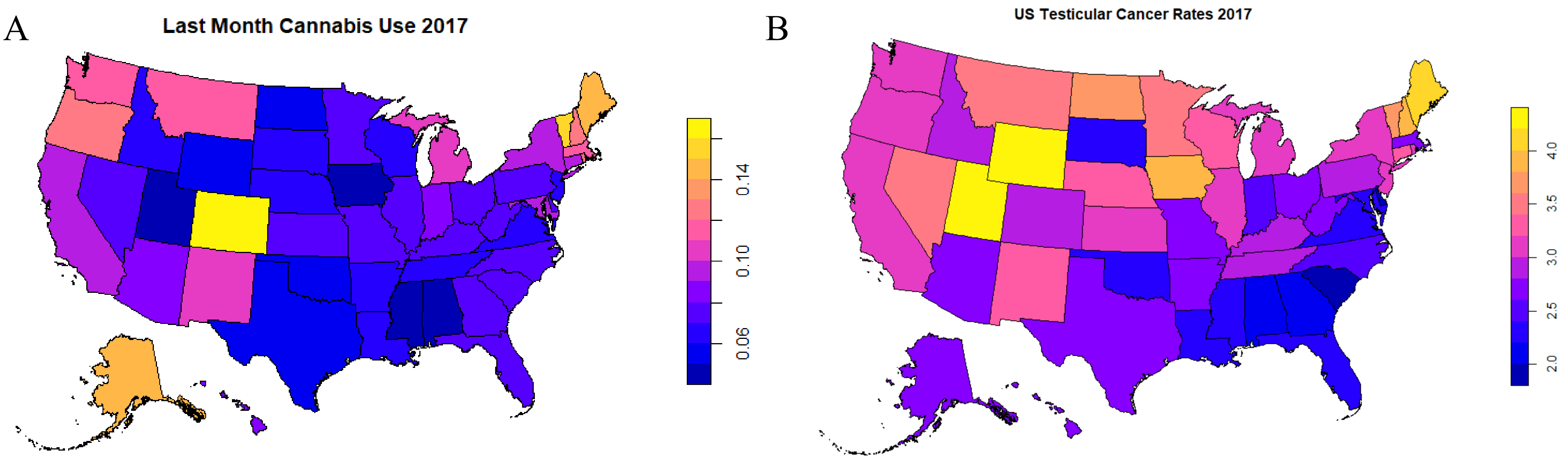

Supplementary Figure Legends

Supplementary Figure S1.: Map of states with available data. (A) Last month cannabis use and (B) testicular cancer incidence rates across USA by state for 2017.
